# Isoniazid improves cognitive performance, clears Aβ plaques, and protects dendritic synapses in APP/PS1 transgenic mice

**DOI:** 10.3389/fnagi.2023.1105095

**Published:** 2023-01-19

**Authors:** Jiacheng Chen, Ning Guo, Yuting Ruan, Yingren Mai, Wang Liao, Yanqing Feng

**Affiliations:** ^1^Department of Neurology, National Key Clinical Department, Guangdong Key Laboratory for Diagnosis and Treatment of Major Neurological Diseases, Guangzhou, China; ^2^Department of Neurosurgery, The First Affiliated Hospital, Sun Yat-Sen University, Guangzhou, China; ^3^Department of Rehabilitation, The Second Affiliated Hospital of Guangzhou Medical University, Guangzhou, China; ^4^Department of Neurology, The Second Affiliated Hospital of Guangzhou Medical University, Guangzhou, China

**Keywords:** Alzheimer’s disease, amyloid β, isoniazid, neuroinflammation, dendritic synapse

## Abstract

**Background and objective:**

Alzheimer’s disease (AD) is characterized by amyloid β (Aβ) aggregation and neuroinflammation. This study aimed to investigate the therapeutic effect of isoniazid (INH) against AD.

**Methods:**

The APP/PS1 transgenic mouse model of AD was adopted. The APP/PS1 mice received oral INH (45 mg/kg/d) for 14 days. The cognitive capability was assessed by the Morris Water Maze test. Amyloid plaques and Aβ levels were determined by immunohistochemistry and ELISA assay. The dendritic spines were analyzed by DiOlistic labeling. Immunofluorescence staining was used to observe the microglia and astrocytes.

**Results:**

The Morris Water Maze test suggested that INH administration can effectively attenuate the reference memory deficit and improve the working memory of the APP/PS1 mice compared to the untreated mice (all *p* < 0.001). INH significantly decreased the Aβ plaques in the hippocampus and cortex and reduced the levels of Aβ_1-40_ and Aβ_1-42_ in the brain homogenates, cerebrospinal fluid, and serum (all *p* < 0.001). INH also inhibited enzyme activities of β-site amyloid precursor protein cleaving enzyme 1 (BACE1, *p* < 0.05) and monoamine oxidase B (Mao-b, *p* < 0.01). INH significantly increased the protrusion density in the hippocampus (*p* < 0.01). Immunofluorescence staining revealed that INH significantly reduced the number of activated microglia and astrocytes around the Aβ plaques (both *p* < 0.01).

**Conclusion:**

Isoniazid administration effectively improved cognitive performance, cleared Aβ plaques, protected dendritic synapses, and reduced innate immune cells around the Aβ plaques, suggesting that INH could be a potential drug for AD treatment.

## 1. Introduction

Alzheimer’s disease (AD) is the most common cause of dementia in the elderly, characterized by a progressive decline of cognitive functions and alterations in the behavior field (Lane et al., 2018). According to the World Alzheimer Report 2016, it was estimated that 46.8 million people worldwide had dementia in 2015, and AD accounts for approximately 60–70% of the cases (Nichols et al., 2019). In addition, the incidence of AD is expected to continue to rise in the next few decades due to the continuous growth of the elderly population (Nichols, 2019). Currently, AD remains an incurable disease and poses a substantial socio-economic challenge. Therefore it is urgently required to develop effective strategies for AD treatment.

The pathogenic mechanisms of AD remain not fully understood. Intracellular neurofibrillary tangles (NFTs) and extracellular neuritic plaque formation are two significant neuropathological changes in AD (Serrano-Pozo et al., 2011). It is well known that the neuritic plaques are associated with the aggregation and deposition of amyloid β peptides (Aβ; Sun et al., 2015). Aβ is composed of 39 to 43 amino acids and has two major isoforms, soluble Aβ_40,_ and insoluble Aβ_42_. Aβ is produced by proteolysis of the transmembrane amyloid precursor protein (APP) by the β-and γ-secretase (Zhang et al., 2012). The β-secretase, also known as β-site amyloid precursor protein cleaving enzyme 1 (BACE1), is the enzyme that initiates the generation of Aβ (Karran et al., 2011). Since accumulation and aggregation of Aβ are considered to be the critical initiator for the progression of AD, Aβ is predicted to be an efficient target for the drug therapies for AD (Vassar, 2004), such as inhibition of the enzymatic activity of BACE1 for reducing cerebral levels of Aβ (Karran et al., 2011).

The innate immune cells-mediated neuroinflammation has also been shown to contribute to the pathogenesis of AD. In the central nervous system, microglia and astrocytes are two major cellular components of the innate immune system and play a crucial role in the neuroinflammation process in AD (Calsolaro and Edison, 2016). During the pathogenesis of AD, both microglia and astrocytes markedly proliferated in an activated form. They gathered around the vicinity of amyloid plaques and NFTs (Calsolaro and Edison, 2016), releasing pro-inflammatory cytokines and chemokines, which lead to neuroinflammation and damage the brain tissue shield (Zhang and Jiang, 2015). Hence, anti-inflammation is also a therapeutic strategy for the treatment of AD.

Isoniazid (INH) is an effective and widely used drug in tuberculosis treatment with the anti-inflammation effect (Sia and Wieland, 2011). It has been shown that INH can be converted into isonicotinamide (Kang et al., 2006), which can inhibit the enzymatic activity of BACE1, thus reducing the generation of Aβ (Stanton et al., 2007). It is well known that INH possesses an excellent therapeutic effect on the central nervous system tuberculosis and can pass easily across the blood–brain barrier, eliciting its impact on the brain (Rock et al., 2008). Based on these notions, we proposed that INH may be a potential drug candidate for AD treatment due to its inhibition effect on BACE1 and its anti-inflammation capacity. However, the impact of INH on AD is still unknown thus far. Therefore, this study aimed to investigate if INH possesses a therapeutic effect against AD by using an APP/PS1 transgenic mouse model of AD.

## 2. Method

### 2.1. Animals

A total of 24 APPswe/PS1dE9 double transgenic mice (Specific Pathogen Free [SPF], 8-month-old, mean weight = 27.70 ± 3.47 g, strain type B6-Tg [APPswe, PS1dE9] 85Dbo/J) and 12 specific-pathogen-free (SPF) 8-month-old wild-type (Wt) mice were purchased from the Model Animal Research Center of Nanjing University (stock number 004462, Nanjing, China). The Institutional Animal Care and Use Committee of Sun Yat-sen University, Guangzhou, China, approved all protocols of this study. The mice were kept in ventilated cages at 25°C and 60% relative humidity room, which has 12:12-h light–dark control (light turned on at 7 AM). The mice can libitum get access to dry food and water.

### 2.2. Isoniazid treatment of model mice

Isoniazid was dissolved by sonication to 2 mg/mL in 0.5% low-viscosity carboxyl methyl cellulose (CMC; Sigma-Aldrich) just before daily use. The 24 APP/PS1 mice were randomly divided into three groups (*n* = 12 for each group). According to the Food and Drug Administration, the oral dose of INH for adults ranges from 100 to 300 mg. Converting from humans to mice, the oral dose would be 20–60 mg/kg, which is equivalent to 1.63–4.86 mg/kg in humans (Nair and Jacob, 2016). INH group that received oral INH (45 mg/kg/d) for 14 days, 450 microlitres of INH solution was orally administered using feeding needles to APPOSK mice, and the Tg Ctrl group that received an equal volume of oral feeding CMC solution. The 12 Wt mice also received an equal volume of CMC solution and designated as the Wt Ctrl group. All animal experiments were approved by the ethics committee of Sun Yat-sen University (Guangzhou, China) and performed by the Guide for Animal Experimentation, Sun Yat-sen University.

### 2.3. Morris water maze

The Morris Water Maze (MWM) device was adopted for the MWM test. A white circular pool with a diameter of 90 cm and a depth of 35 cm, equally divided into four quadrants and filled with water (22°C), was used. A submerged platform (10 cm diameter and 25 cm in height) painted in black was placed in 1 of 4 quadrants and submerged 1 cm below the water surface. The place navigation trial and spatial probe trial were included in the MWM test, which aimed to test mice’s reference memory and working memory. The test consisted of an acquisition trial for 5 days, a one-day extinction phase, and a 2-day reversal phase (Vorhees and Williams, 2006). The paradigm consisted of four shots per day during the acquisition trial. In each test, the mice were placed at one of four different start positions. The timer was set to 60 s and automatically stopped once the mouse reached the platform within 60 s and remained on the platform for 5 s. If not, the mouse was guided to the forum and on it for 20 s. At 24 h after the last day of spatial training, the medium was removed, and the probe trial was carried out, allowing each mouse to swim for 60 s. Finally, a 2-day trial of reversal phase (four trials/day) was performed with the platform located in the opposite quadrant.

### 2.4. Sample collections

After the MWM, the mice were anesthetized with urethane (25%, 0.5 ml/100 g). At first, the cerebrospinal fluid (CSF) was collected by a cauda equine catheterization technique. The whole procedure of cauda equine catheterizations was performed under a dissecting microscope. A 2-cm longitudinal incision was made on the skin at the spine between the femoral heads. The spinous processes were exposed with blunt separation, and a pair of toothed forceps was used to pull out the spinous process slowly. The cauda equine was then exposed, and the spinal dura mater could be seen. A V-shaped incision was made on the spinal dura mater, and the cauda equine could be seen. The spinal dura mater was lifted and the cannula was inserted into the subarachnoid space. After inserting the equine cauda cannula, CSF flowed into a 1.5 ml Eppendorf tube. The terminal of the cannula should be snipped into a bevel and adhered to the wall so that the mice’s intracranial pressure would not exceed the surface tension. After 2 ~ 3 h of collection, the volume of CSF can reach 100–200 μl. Then the blood sample was collected by heart puncture and placed at room temperature (25°C) for 2–3 h for sedimentation. After centrifugation at 1,000×*g* for 15 min at 4°C, the serum was collected and stored at –80°C until further use. For ELISA and BACE1 activity assay, the brains were taken directly after perfusion by physiological saline with 1% heparin and stored at-80°C. For immunohistochemistry and DiOlistic labeling, the brains were removed after intracardially perfused with 0.9% saline, followed by 4 and 2% paraformaldehyde in a 0.1 M phosphate buffer (pH 7.4), respectively.

### 2.5. Elisa

Aβ_1-40_ and Aβ_1-42_ levels in brains, serum, and cerebrospinal fluid (CSF) of APP/PS1 mice were measured with Aβ_1-40_ and Aβ_1-42_ ELISA kit (KHB3481 and KHB3441, Invitrogen, United States), respectively. Protein concentration was measured by Pierce™ BCA Protein Assay Kit (Catalog: 23225, Thermo Fisher Scientific). Briefly, the brain tissue was homogenized in PBS (0.01 M) with 1 mM EDTA, 1% BSA (Sigma, United States), 2% Triton X-100 and protease inhibitor cocktail (Sigma) at 14000× g for 30 min at 4°C. The supernatant was collected for the assay of soluble proteins. The insoluble pellet was dissolved in guanidine hydrochloride and centrifuged at 1,00,000×*g* for 1 h at 4°C, and the resulting supernatant was collected for the assay of insoluble protein. The ELISA assay has been extensively tested, and no cross-reactivity between Aβ_1-40_ and Aβ_1-42_ was found.

### 2.6. β-site amyloid precursor protein cleaving enzyme 1 enzymatic activity

β-site amyloid precursor protein cleaving enzyme 1 enzymatic activity was analyzed using the β-Secretase Activity Assay Kit, Fluorogenic (Catalog: 565785, Calbiochem), according to the manufacturer’s protocol. The fluorescence intensity was measured with a microplate reader (Sunrise, TECAN) at excitation wavelength 350 nm and emission wavelength 510 nm.

### 2.7. Monoamine oxidase activity

The brains of mice were homogenized as previously described (Naoya Aoo, 2013) and the protein concentrations of the homogenates were measured as above. Monoamine oxidase (MAO) activity was determined with the Amplex Red Monoamine Oxidase Assay Kit (A12214, Molecular Probes, OR, United States). The homogenates were mixed in equal amounts with a working solution composed of Amplex Red (200 μM), horseradish peroxidase (1 U/mL), and MAO substrate (p-tyramine or benzylamine; 1 mM) and incubated at 37°C for 1 h. MAO-B activity was detected using benzylamine for the MAO-B substrate. The fluorescence intensity was measured using a fluorescence plate reader (SpectraMax M5, Molecular Devices, United States) at an excitation wavelength of 535 nm and an emission wavelength of 590 nm.

### 2.8. Immunohistochemistry

The brains were fixed with 4% paraformaldehyde in 0.1 M phosphate buffer (pH 7.4) at 4°C for 48 h and then cryoprotected in 30% (w/v) sucrose in 0.01 M PBS. Using a microtome, the brains were sectioned into 20-μM thick coronal sections (Leica, Germany). Amyloid pathology was assessed using mouse monoclonal anti-β-Amyloid antibody (1:500; A3981; Sigma), followed by a biotinylated secondary antibody (Invitrogen) for 1 h at room temperature and visualized using DAB development according to manufacturer’s protocol. Stained slides were examined by light microscopy (BX63, Olympus, Germany), six sections from each mouse were used for quantification analysis. For Aβ plaque, the surface area of the plaques was measured and compared as the percentage of the dentate gyrus with Image-Pro plus 6.0 (Media Cybernetics, Inc., Bethesda, Maryland, United States).

### 2.9. Double immunofluorescence staining

To assess the Aβ plaque-associated microglia and astrocytes, double staining of GFAP/Aβ and Iba1/Aβ was performed. The primary antibodies against GFAP (1:500, rabbit, Sigma G4546,), Aβ (1:500; mouse, Sigma, cat no. A3981) and IBA1 (1:500; rabbit, Wako Chemical, United States; cat no. 01919741) were used, followed by incubated with Alexa Fluor 647-conjugated anti-mouse IgG secondary antibody (1:1000, Cell signaling, cat no. 4410), appropriate secondary antibodies (Alexa Fluor 568-labeled anti-rabbit IgG, Invitrogen A11036) at 1:200. Finally, 4′,6-diamidino-2-phenylindole (DAPI; 10,236,276,001, Sigma) was added as a nuclear dye in the immunofluorescence staining slide. The slide was visualized by a Zeiss LSM 780 laser confocal scanning microscope (Carl Zeiss AG, Oberkochen, Germany). To obtain morphological measures of Aβ-associated microglia and astrocytes, a circular Region of Interest (ROI, 75-μM-diameter) was centered over the plaque to define plaque domains and then superimposed on images of Iba1-stained microglia and GFAP-stained astrocytes. Total Aβ-associated microglia and astrocyte density were calculated within each plaque domain, with a distinct cell soma required for cell counts. Iba1 and GFAP fluorescence intensities were also calculated within each plaque domain.

### 2.10. DiOlistic labeling

The brains processed as above were coronally sectioned into 30-μM-thick sections with a vibratome. The gene gun bullets were prepared as previously described (Staffend and Meisel, 2011). Then the gene gun bullets were delivered biolistically into tissue at 170 psi using a gene gun tubing (Bio-Rad, Hercules, CA, United States). The labeled sections were rinsed in 0.01 M PBS for 3 times. Then DiI dye was allowed to diffuse along neuron dendrites and axons with the labeled sections in PBS at 4°C overnight, followed by DAPI staining. The images of DiI impregnated cells were taken using the Zeiss LSM 780 confocal microscope. DiI was excited using the Helium/Neon 543 nm laser line. The neuron was scanned at 1 μM intervals along the z-axis and the topology of the dendritic tree was reconstructed in 3-D LAS AF software (Leica Microsystems, Buffalo Grove, IL, United States). The 3D reconstructions of confocal images were performed with an Imaris 6.4.2 (Bitplane, Zurich, Switzerland). The density of the dendritic spines was measured on 3–4 randomly chosen dendrites from each neuron, calculated by the number of spines per unit length of dendrite and normalized per 10 μM of dendrite length.

### 2.11. Statistical analysis

Continuous data were presented as the mean ± standard deviation (SD). The MWM data was analyzed using two-way mix designed ANOVA with repeated measures and Fisher’s LSD comparison as a post-hoc test if overall significance was found in ANOVA. In the analyses of ELISA, immunohistochemistry assays, immunofluorescence assays, DiOlistic labeling, one-way ANOVA was used to determine the difference among four groups and Fisher’s LSD comparison as a post-hoc test. The statistical significance level for all the tests was set at a *p*-value <0.05. All analyses were performed using SPSS software (SPSS Statistics V20, IBM Corporation, Somers, New York, United States).

## 3. Results

### 3.1. Isoniazid improved the spatial learning abilities of the APP/PS1 mice in the Morris Water Maze

**Figure 1:**
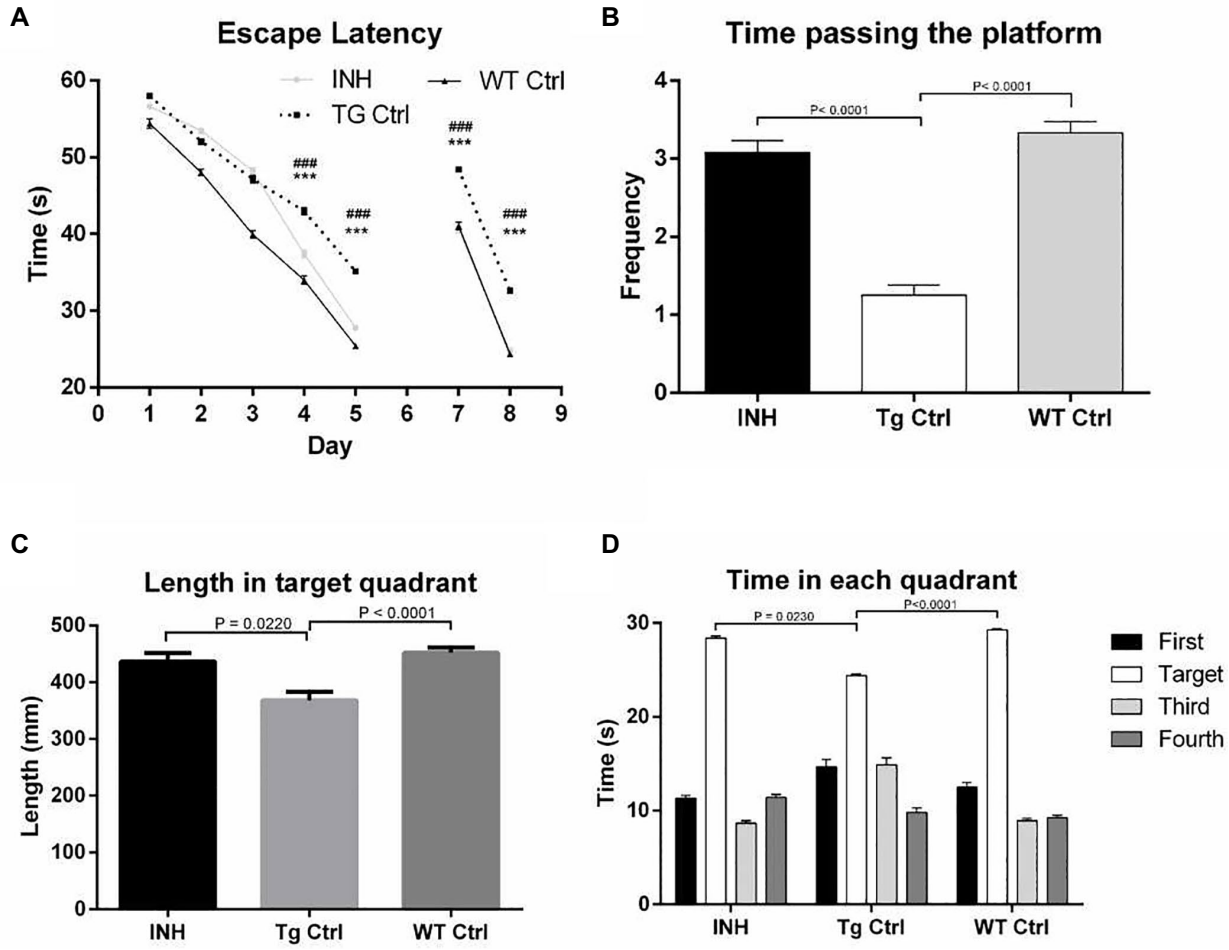
INH improved the spatial learning abilities of the APP/PS1 mice in the MWM. **(A)** Comparison of the escape latency time in the learning trail and the reversal test. In the probe trial, the times of platform crossing **(B)**, the path length at the target quadrant **(C)**, and the time spent in the target quadrant **(D)** were compared among the groups.

To assess whether INH could improve the cognitive abilities of APP/PS1 mice, MWM test was performed. In the learning trail, compared to the Wt Ctrl group, the Tg Ctrl group had longer escape latency times at day 4 and day 5 (Figure 1A). However, INH administration significantly reduced the escape latency times in APP/PS1 mice at day 4 and day 5 as compared with the Tg Ctrl group (all *p* < 0.001). In the probe test, the INH group had more times of platform crossing (Figure 1B), increased path length at the target quadrant (Figure 1C) and more time spent in the target quadrant (Figure 1D) as compared with the Tg Ctrl group (all *p* < 0.001). These results suggested that INH can effectively attenuate the reference memory deficit of the APP/PS1 mice. In the reversal test, INH group had significantly shorter escape latency times at day 7 and day 8 than the Tg Ctrl group, indicating that INH can improve the working memory of the APP/PS1 mice (both *p* < 0.001, Figure 1A).

### 3.2. Isoniazid decreased the level of Aβ in the brain of APP/PS1 mice

**Figure 2:**
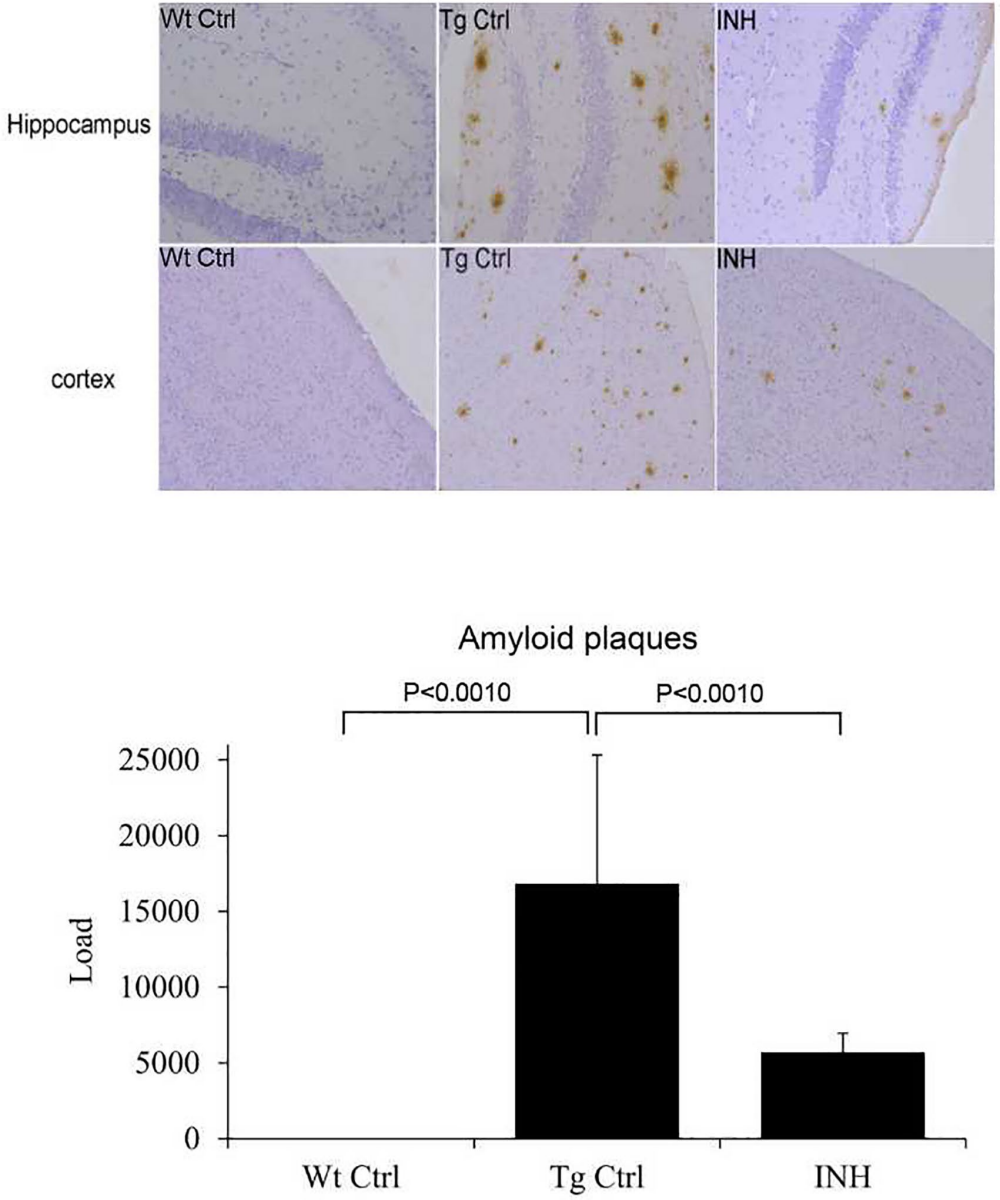
INH decreases Aβ plaques in the brains of APP/PS1 mice. The representative images of IHC staining for Aβ1-42 in the hippocampus and cortex were shown. The bar chart showed the quantitative result of Aβ plaques in the IHC images.

Since INH treatment can improve learning and memory capability of APP/PS1 mice, we then attempted to investigate the mechanism underlying the protective effect of INH. To address if INH could alter the morphological change in the brain of APP/PS1 mice, IHC staining was performed. As shown in Figure 2, the Aβ plaques in the hippocampus and cortex was significantly decreased in the INH group as compared with the Tg Ctrl group (*p* < 0.001).

**Figure 3:**
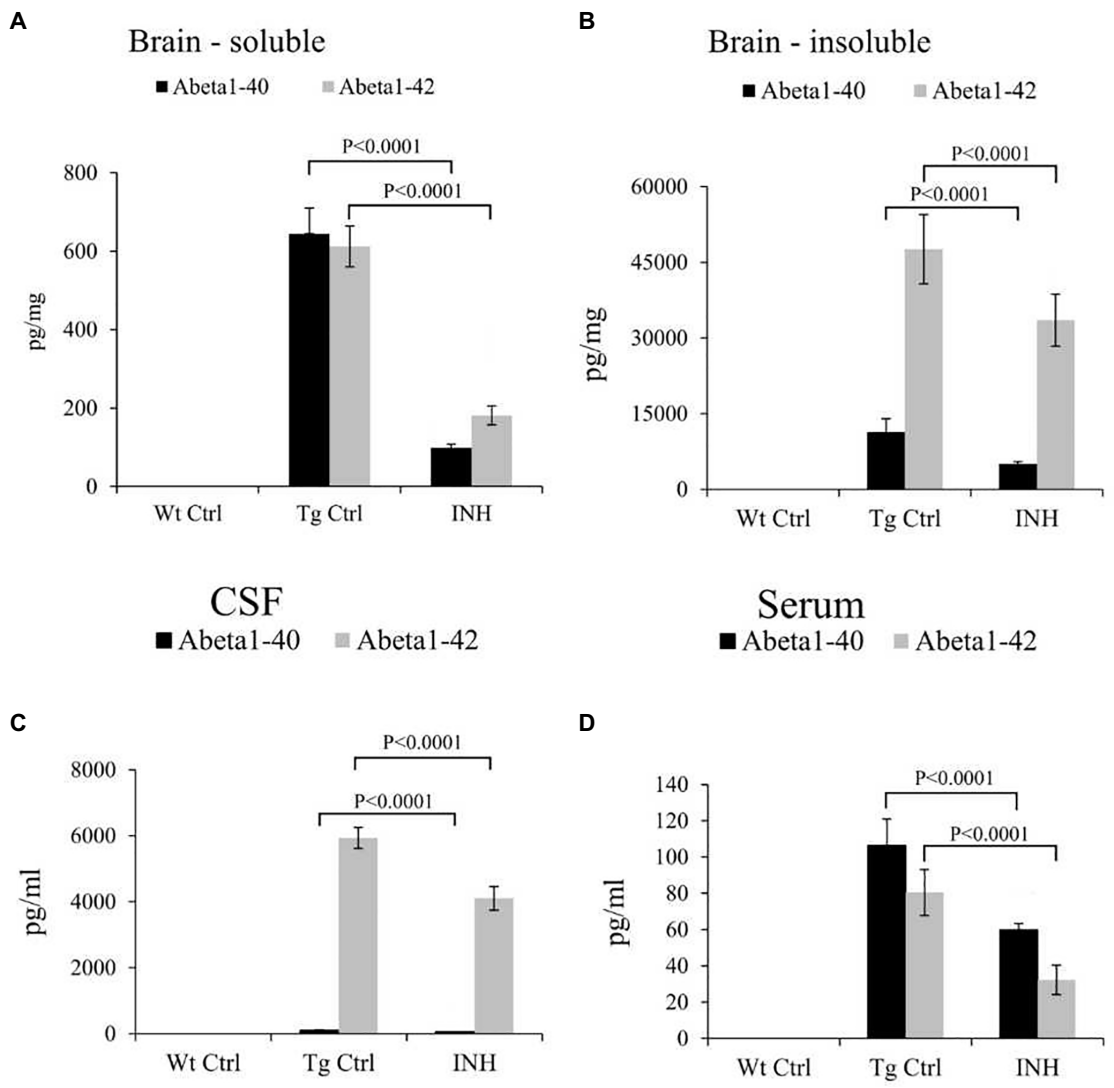
INH reduced the levels of Aβ in APP/PS1 mice. The total levels of soluble Aβ1-40 and Aβ1-42 **(A)**, as well as insoluble Aβ1-40 and Aβ1-42 **(B)** in the brain homogenates, were measured by ELISA assay. The levels of soluble Aβ1-40 and Aβ1-42 in the CSF **(C)** and serum **(D)** were also determined.

In addition, ELISA analysis revealed that the INH group had significantly lower levels of Aβ_1-40_ and Aβ_1-42_ in the soluble (Figure 3A) and insoluble (Figure 3B) brain homogenates (all *p* < 0.001), as well as the Aβ_1-40_ and Aβ_1-42_ levels in the cerebrospinal fluid (CSF, Figure 3C) and the serum (Figure 3D) as compared with the Tg Ctrl group (all *p* < 0.001).

### 3.3. Isoniazid inhibited the enzyme activities of β-site amyloid precursor protein cleaving enzyme 1 and monoamine oxidase B in APP/PS1 transgenic mice

**Figure 4:**
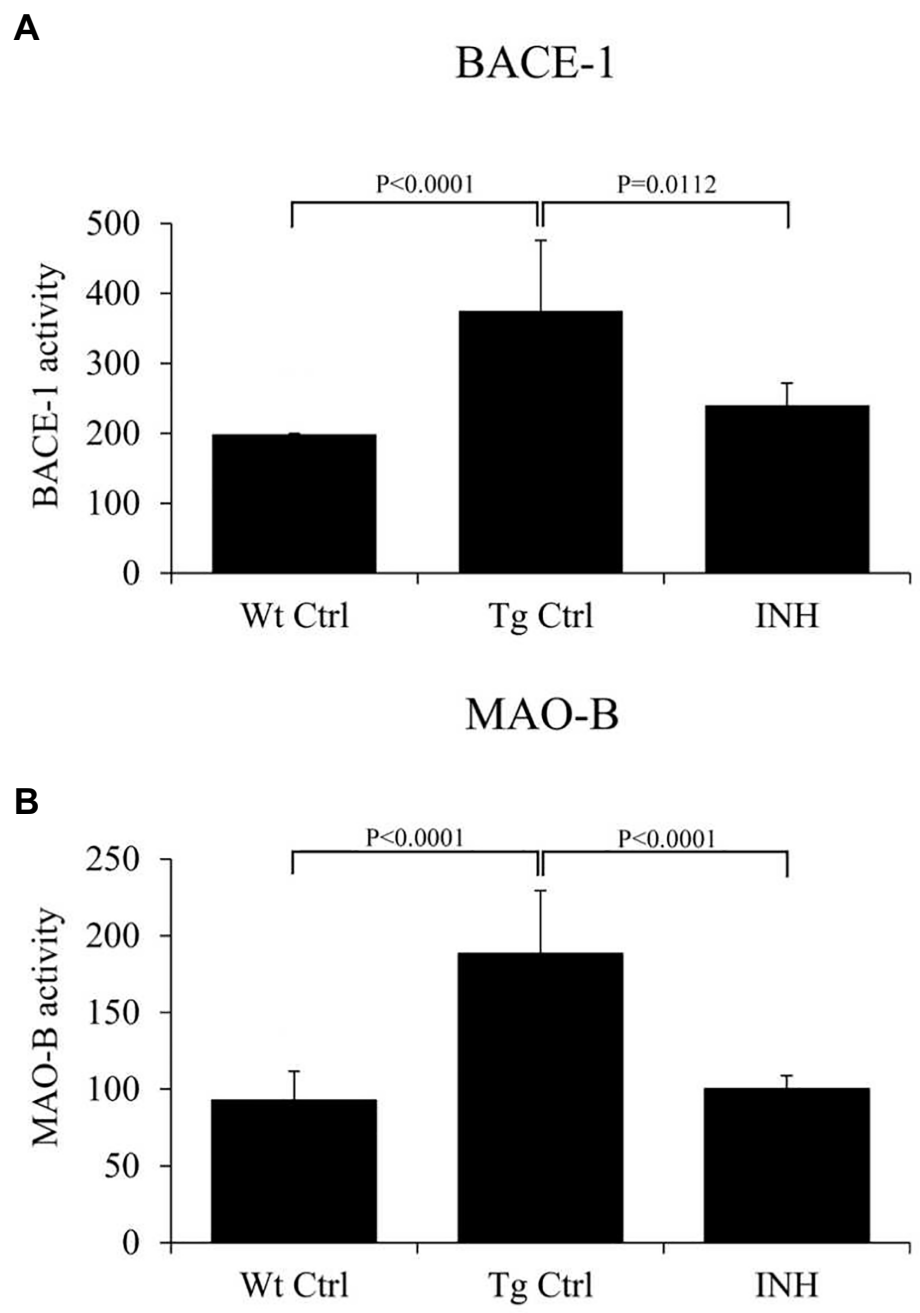
**(A,B)** INH inhibited the enzyme activities of BACE1 and Mao-b in the hippocampus of APP/PS1 mice.

β-site amyloid precursor protein cleaving enzyme 1 is the key enzyme in the production of β-amyloid (Kandalepas and Vassar, 2014), and monoamine oxidase B (Mao-b) can regulate Aβ production (Schedin-Weiss et al., 2017). It has been shown that BACE1 and Mao-b activity were significantly elevated in APP/PS1 mice (Jo et al., 2014). Since INH treatment significantly reduced the level of Aβs, we further investigate if INH affects the enzyme activities of BACE1 and Mao-b in the mice brain. The results showed that INH significantly reduced the enzyme activities of BACE1 (*p* < 0.05, Figure 4A) and Mao-b (*p* < 0.01, Figure 4B).

### 3.4. Effect of isoniazid on synaptic protection

**Figure 5:**
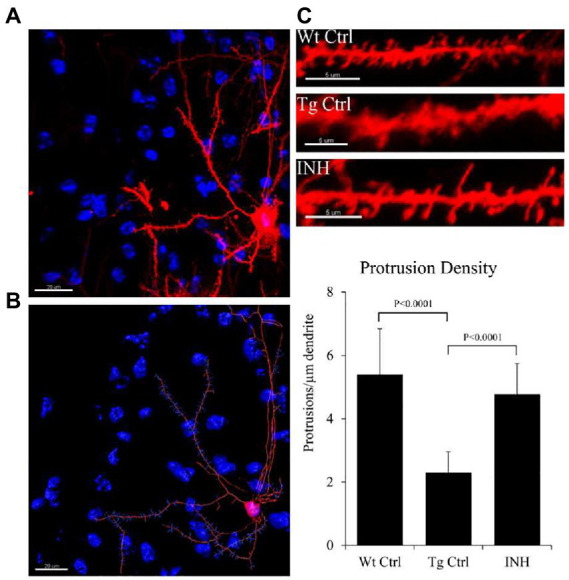
**(A–C)** INH increases the density of dendritic spines in the brains of APP/PS1 mice. Representative images of the DiOlistic labeling of a single neuron from the brains of each group were shown. The bar chart showed the quantitative result of the density of dendritic spines.

To investigate if INH treatment has an effect on the synaptic changes in dendrites, DiOlistic labeling was used to observe the dendritic spines of neurons in the dentate gyrus and cortex of the hippocampus. Confocal images of the hippocampus and DiI-labeled dendrite segments from the brain slices were shown in Figure 5. The quantitative result showed that the mean number of dendrites in each neuron (protrusion density) significantly increased in the INH group as compared with the Tg Ctrl group (*p* < 0.01, Figure 5).

### 3.5. Isoniazid reduced the microglial and astrocytes surrounding the Aβ plaques

**Figure 6:**
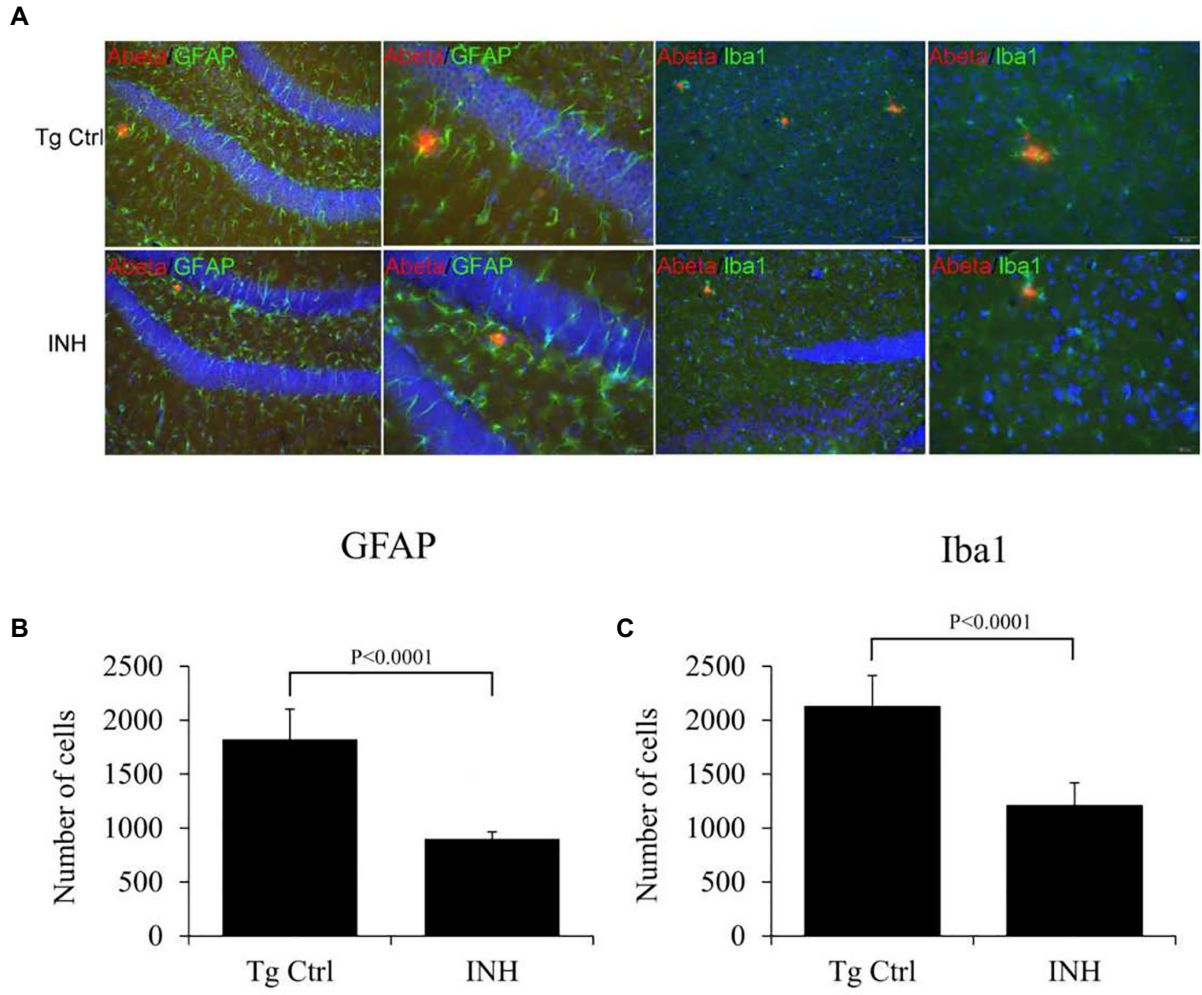
**(A–C)** INH reduced the microglial and astrocytes activation. Representative confocal images showed the Aβ plaques (red), microglial cells (IBa1+ cells, green), and activated astrocytes (GFAP+ cells, green) in the subgranular zone of the hippocampus of the INH group and the Tg Ctrl group. The numbers of microglia or activated astrocytes around the Aβ plaques were quantitated. **p < 0.01, compared with the Tg Ctrl group.

Next, we investigated if the beneficial effect of INH is implicated in the neuroinflammation in the brain of APP/PS1 mice. Both microglia (Xiao et al., 2016) and astrocytes (Velázquez-Moctezuma et al., 2014) have been identified as the inflammatory components in the pathogenesis of AD. Thereby immunofluorescence staining was conducted to determine the numbers of microglia (IBa1^+^ cells, green color) or activated astrocytes (GFAP^+^ cells, green color) around the Aβ plaques (red color, Figure 6A) in the subgranular zone of the hippocampus. The quantitative results showed that the number of GFAP^+^ cells (Figure 6B) and IBa1^+^ cells (Figure 6C) around the Aβ plaques were significantly reduced in the INH group as compared with the Tg Ctrl group (both *p* < 0.01).

## 4. Discussion

In this study, we investigated the therapeutic effect of INH against AD in an APP/PS1 transgenic mouse model. The Morris Water Maze test suggested that INH oral administration can effectively attenuate the reference memory deficit and improve the working memory of the APP/PS1 mice. In addition, INH significantly decreased Aβ plaques in the hippocampus and cortex and reduced the levels of Aβ_1-40_ and Aβ_1-42_ in the brain homogenates, CSF and the serum. INH significantly reduced the enzyme activities of BACE1 and Mao-b. Confocal images showed that INH significantly increased the protrusion density in the hippocampus. Moreover, immunofluorescence staining revealed that INH significantly reduced the number of activated microglia and astrocytes around the Aβ plaques. Taken together, these results suggested INH possesses the therapeutic effect against AD. To our best knowledge, this is the first study reporting the therapeutic effect of INH against AD. Although INH treatment has been reported to increase hepatotoxicity and peripheral neurotoxicity (Preziosi, 2007), the adverse effects can be avoided by reducing drug dose or supplementation with vitamin B6 (Rasul and Das, 2000). In this study, the daily dose of INH administration (45 mg/kg) was determined according to the clinical dose to prevent hepatotoxicity.

In this study, MWM results showed that INH group had shorter escape latency times and more times of platform crossing in probe trials, indicating that INH administration can effectively improve the spatial and related forms of learning and memory of APP/PS1 mice. To investigate the mechanism of the protective effect of INH, we further assessed the levels of Aβ plaques and Aβ in the brain and serum. IHC and ELISA data demonstrated that INH treatment significantly decreased the levels of senile plaque deposits as well as Aβ_1-40_ and Aβ_1-42_ in the APP/PS1 mice. In addition, further investigation showed that INH significantly inhibited BACE1 activities in APP/PS1 mice. This may be attributed to the reason that INH could be converted into isonicotinamide (Kang et al., 2006) to inhibit the enzymatic activity of BACE1. However, the details mechanism of inhibition effect of INH on BACE1 activity remains to be further elucidated.

Mao-b is predominantly expressed in astrocytes in the hippocampus (Saura et al., 1992). Alterations in MAO activity have been implicated in several nervous disorders (Pintar and Breakefield, 1982). Furthermore, Mao-b-mediated putrescine degradation pathway is responsible for GABA production in activated astrocytes, which plays an important role in impairing memory of APP/PS1 mice (Jo et al., 2014). Mao-b has also been shown to play a role in the regulation of Aβ production (Schedin-Weiss et al., 2017). Our result showed that Mao-b activity significantly elevated in APP/PS1 mice as compared with wild-type mice, which is consistent with the previous finding that Mao-b is elevated in the brain neurons of AD patient (Schedin-Weiss et al., 2017). In addition, our result showed that INH treatment considerably inhibited Mao-b activity in APP/PS1 mice. Interestingly, the Mao-b inhibitors, such as selegiline, have been shown to possess the beneficial effects on cognitive impairment in AD patients (Burke et al., 1993; Tolbert and Fuller, 1996; Filip and Kolibás, 1999). Therefore the inhibitory effect of INH on Mao-b may also contribute to the therapeutic effect on memory impairment in APP/PS1 mice. Further study should be conducted to investigate the detailed mechanism.

Dendritic spines are specialized structures on neuronal processes cells and are essential for excitatory synaptic transmission (Bonhoeffer and Yuste, 2002). It has been shown that APP/PS1 mice have reduced spine density in CA1 pyramidal neurons which leads to impairment of synaptic connectivity and is associated with cognitive impairment (Perez-Cruz et al., 2011). The abnormal changes of dendrites are related to a decline in synaptic plasticity and neuron impairment, which is mainly caused by the increasing level of oxidative stress and Aβ aggregation (Moolman et al., 2004). In this study, we also observed that the dendritic spine density was significantly decreased in APP/PS1 mice, whereas INH treatment significantly elevated the dendritic spine density. It is worth to further investigate if the reduction of Aβ aggregation accounts for the synaptic protection effect of INH.

Neuroinflammation is a well-recognized feature and a potential target for therapy and prevention of AD (Zhang and Jiang, 2015). In the AD brain, Aβ can trigger neuroinflammation by activating microglia to release pro-inflammatory cytokine and inflammatory mediators (Szczepanik and Ringheim, 2003). Although early microglial recruitment can promote Aβ clearance and hinder the pathologic progression in AD, a persistent microglial accumulation leads to successive release pro-inflammatory cytokines, chronic neuroinflammation and eventual neuronal damage (Hickman et al., 2008). Our result showed that INH treatment significantly reduced the number of activated microglia and astrocytes around the Aβ plaques, implying that neuroinflammation was attenuated in INH-treated animals. This effect may be associated with the anti-inflammation effect of INH. Nevertheless, the mechanism requires further investigation.

There are still some limitations of this study. Firstly, the detailed molecular mechanism underlying the protective effect of INH against AD should be further elucidated. Secondly, although we found that INH reduced innate immune cells around Aβ plaques, we did not determine the levels of pro-inflammation cytokines and chemokine to evaluate the status of neuroinflammation. In addition, short-term administration of INH might influence the result interpretation. All these limitations should be addressed in the following study. Overall, the study provides some inspiring data and indicates INH administration effectively improved cognitive performance, cleared Aβ plaques, protected dendritic synapses and reduced innate immune cells around the Aβ plaques in APP/PS1 mice, suggesting that INH could be a potential drug for AD treatment. Orally available, little adverse effects, and good efficacy.

In conclusion, this study has identified, for the first time, the potential efficacy of INH against AD: i.e. orally available, little adverse effects, and good efficacy at improving cognitive performance, cleared Aβ plaques. Thus, INH could be a promising, ready-to-use medicine for the prevention of Alzheimer’s disease. Additional efforts are necessary on the interaction between INH and protein oligomers, and well-designed clinical trials should be conduct since no other effective treatments currently exist to reverse AD progression.

## Data availability statement

The original contributions presented in the study are included in the article/supplementary material, further inquiries can be directed to the corresponding authors.

## Author contributions

YF and WL conceived and designed the experiments. JC, NG, and YF wrote the article. All authors contributed to the article and approved the submitted version.

## Funding

This work supported by National Natural Science Foundation of China (No. 82101271). This work was also supported by grants from the Key Clinical Department, National Key Discipline, Guangdong Key Laboratory For Diagnosis and Treatment of Major Neurological Diseases (No. 2010A060801005), Guangdong Science and Technology Project (No. 2013B051000018), and Guangdong Basic and Applied Basic Research Foundation (Nos. 2020A1515110317 and 2021A1515010705).

## Conflict of interest

The authors declare that the research was conducted in the absence of any commercial or financial relationships that could be construed as a potential conflict of interest.

## Publisher’s note

All claims expressed in this article are solely those of the authors and do not necessarily represent those of their affiliated organizations, or those of the publisher, the editors and the reviewers. Any product that may be evaluated in this article, or claim that may be made by its manufacturer, is not guaranteed or endorsed by the publisher.
